# Plasma and serum concentrations of VEGF-A121, but not of VEGF-A165, increase post-bevacizumab administration

**DOI:** 10.1371/journal.pone.0316035

**Published:** 2024-12-19

**Authors:** Masashi Okawa, Munekazu Yamakuchi, Aryal Bibek, Kazunori Takenouchi, Drew N. Maywar, Shingo Yamada, Keiichi Inoue, Kazuhiko Higurashi, Junichi Nakazawa, Masahiro Kawahira, Tomoko Kodama, Kiyonori Tanoue, Yoko Oyama, Sadayuki Higashi, Chieko Fujisaki, Hirohito Hashinokuchi, Akito Tabaru, Hideaki Kanda, Shuji Tachioka, Yutaka Imoto, Teruto Hashiguchi, Yoshiharu Soga

**Affiliations:** 1 Department of Cardiovascular and Gastroenterological Surgery, Graduate School of Medical and Dental Sciences, Kagoshima University, Kagoshima, Japan; 2 Department of Laboratory and Vascular Medicine, Graduate School of Medical and Dental Sciences, Kagoshima University, Kagoshima, Japan; 3 Kagoshima University Hospital Clinical Laboratory, Kagoshima, Japan; 4 Electrical and Computer Engineering Technology, Rochester Institute of Technology, Rochester, New York, United States of America; 5 Shino-Test Corporation, Sagamihara, Japan; 6 Department of Medical Oncology, Kagoshima City Hospital, Kagoshima, Japan; Hokkaido University: Hokkaido Daigaku, JAPAN

## Abstract

**Background:**

VEGF-A concentrations were measured in the blood of bevacizumab-treated cancer patients in previous studies, but a consensus has not formed that would develop VEGF-A into a clinical biomarker. Recently, methods to strictly distinguish between the VEGF-A isoforms have been developed but have not yet been applied to cancer patients undergoing bevacizumab treatment.

**Methods:**

An ELISA that strictly distinguishes between VEGF-A121 and VEGF-A165—the major isoforms of VEGF-A—and a commercially available ELISA for VEGF-A are used to determine the concentration of VEGF-A121, VEGF-A165, and VEGF-A in the blood of 12 patients with advanced colorectal cancer receiving bevacizumab therapy.

**Results:**

The serum and plasma concentrations of VEGF-A121 increased substantially post-bevacizumab administration; the median increase in serum was 860.8 pg/mL, 95% confidence interval (CI) [468.5, 1128.9], *p* = 0.0024, and in plasma was 808.6 pg/mL, 95% CI [748.7, 874.0], *p* = 0.00049. In stark contrast, VEGF-A165 after bevacizumab administration decreased in serum by a medium change of –73.8 pg/mL, 95% CI [–149.4, –10.2], *p* = 0.0034, with 83.3% of the post-bevacizumab concentrations falling below the high-accuracy threshold of 38 pg/mL; in plasma, all pre and post VEGF-A165 concentrations fell below this threshold. Concentrations of VEGF-A121 and VEGF-A165 in platelets did not change to a statistically significant degree. Adding recombinant VEGF-A121 (and -A165) or bevacizumab to plasma in patients post-bevacizumab administration increased or decreased, respectively, VEGF-A121 and VEGF-A165 levels. The increase in VEGF-A121 in plasma and serum after bevacizumab administration may be due to the dissociation of the complex of tumor-derived VEGF-A121 and bevacizumab when it moves from the stroma into the blood.

**Conclusions:**

The VEGF-A121 isoform has been uniquely demonstrated as a clear marker of bevacizumab therapy in both plasma and serum, motivating further research on pursuing these isoforms as biomarkers in cancer care.

## Introduction

Targeting of VEGF-A, a key regulator of normal and pathological angiogenesis, has provided innovative cancer therapies [1]. The recombinant humanized VEGF-A-specific monoclonal antibody, bevacizumab (Avastin; Genentech/Roche), was approved in 2004 by the US Food and Drug Administration for the first-line treatment of metastatic colorectal cancer [2]. An important question in anti-VEGF-A therapy is the identification of patients who could benefit the most from this therapy [1]. In many clinical trials, tumor and plasma VEGF-A concentrations were measured, but these could not be translated into biomarkers for clinical use [3–8]. To date, there is no consensus on the kinetics of VEGF-A post-bevacizumab administration, with some reports showing a decrease post-administration [9–11] and others showing an increase [9, 12–17].

The human VEGF-A gene, *VEGFA*, is comprised of eight exons. Multiple isoforms of VEGF-A, derived from the alternative splicing of exons 6 and 7, are known; these are VEGF-A121, VEGF-A165, VEGF-A189, and VEGF-A206 [18]. VEGF-A189 and VEGF-A206 have a heparin-binding domain encoded by exons 6 and 7, which explains the strong binding of these isoforms to heparan sulfate proteoglycan (HSPG) in the extracellular matrix (ECM) [19]. In contrast, VEGF-A121 lacks the heparin-binding domain and can therefore diffuse freely [19]. VEGF-A165 has intermediate ECM-binding, owing to its sequence encoded by exon 7, and has optimal bioavailability and bioefficacy [19]. Thus, there are multiple splicing variants of VEGF-A, distributed nonlinearly in tissues and blood [20]. Furthermore, the two most major isoforms, VEGF-A121 and VEGF-A165, have opposing effects in the tumor environment, increasing the need for assays that strictly distinguish between them [21].

Most commercially available ELISA kits (e.g., Human VEGF Quantikine ELISA Kit, R&D Systems, Minneapolis, MN) for quantitation of human VEGF-A concentrations detect both VEGF-A121 and VEGF-A165. We, therefore, generated two monoclonal antibodies specific to VEGF-A121 or VEGF-A165 and developed ELISA methods using these antibodies [22]. These new methods exhibit a working range of 0 to 2000 pg/mL and high-accuracy thresholds of 35 and 38 pg/mL for VEGF-A121 and VEGF-A165, respectively [22]; below these thresholds, the methods produce results but with less accuracy.

These new methods of VEGF-A121 and VEGF-A165 quantitation were proven out with samples from healthy individuals [22]. In the present paper, we apply these methods to quantitate VEGF-A isoforms in blood obtained from patients with metastatic colorectal cancer undergoing bevacizumab treatment. Doing so provides evidence for these isoforms as markers in cancer treatment.

## Materials and methods

### Patients and bevacizumab treatment

This study was approved by the research ethics committees of Kagoshima University Hospital and Kagoshima City Hospital (approval numbers 190067, 2019–38). The start and end of the recruitment period for this study is November 22, 2019 and February 8, 2021. All the patients provided written informed consent prior to inclusion in the study. The study was conducted in accordance with the ethical standards of the Committee on Human Experimentation of the institution at which the experiments were performed or in accordance with the ethical standards prescribed in the Helsinki Declaration of 1975. Blood samples were obtained from 12 patients with colorectal cancer who were initially administered bevacizumab treatment. All the patients were first-time bevacizumab recipients. Blood samples were collected immediately pre-bevacizumab administration, 2–4 weeks post administration, and before the second course of administration, either by peripheral venous blood sampling or blood sampling through a subcutaneous implant port. The patient characteristics and clinical features are listed in Table 1.

**Table 1:** The characteristics and clinical features of 12 patients.

Patinent	Sex	Age	Tumor sites	Metastatic lesion	UICC TNM claassification	Chemotherapy regimen	Dose of Bevacizumab	Schedule
1	Female	72	Rectum	Liver	Stage Ⅳ	Cap+Bevacizumab	7.5mg/kg	every 3 weeks
2	Male	75	Transverse colon	Liver	Stage Ⅳ	Cap+Bevacizumab	7.5mg/kg	every 3 weeks
3	Female	47	Ascending colon	Liver	Stage Ⅳ	CapeOX+Bevacizumab	7.5mg/kg	every 3 weeks
4	Female	58	Rectum	Bone	Stage Ⅳ	FOLFOXIRI+Bevacizumab	5mg/kg	every 2 weeks
5	Male	65	Rectum	Lymph node	Stage Ⅳ	CapeOX+Bevacizumab	7.5mg/kg	every 3 weeks
6	Male	68	Sigmoid colon	Lung	Stage Ⅳ	FL+Bevacizumab	5mg/kg	every 2 weeks
7	Male	36	Rectum	Lung, Lymph node	Stage Ⅳ	TAS102+Bevacizumab	5mg/kg	every 4 weeks
8	Female	57	Rectum	Uterus, Lymph node	Stage Ⅳ	IRIS+Bevacizumab	5mg/kg	every 4 weeks
9	Male	33	Rectum	-	Stage ⅢC	mFOLFOXIRI+Bevacizumab	5mg/kg	every 2 weeks
10	Male	59	Rectum	Liver	Stage Ⅳ	mFOLFOXIRI+Bevacizumab	5mg/kg	every 2 weeks
11	Male	71	Sigmoid colon	-	Stage ⅢC	CapeOX+Bevacizumab	7.5mg/kg	every 3 weeks
12	Male	75	Sigmoid colon	Liver	Stage Ⅳ	Cap+Bevacizumab	7.5mg/kg	every 3 weeks

UICC, Union for International Cancer Control, capecitabine; CapeOX, capecitabine and oxaliplatin; FOLFOXIRI, 5-fluorouracil, leucovorin, oxaliplatin and irinotecan; FL, 5-fluorouracil and leucovorin; IRIS, irinotecan and S-1; mFOLFOXIRI, modified FOLFOXIRI

### Serum and plasma preparation

Whole blood was collected in a serum separating tube (Venoject II, Terumo Corp, Tokyo, Japan) and a citrate tube containing 3.2% sodium citrate (Venoject II, Terumo Corp.) for plasma collection. The serum separating tube was incubated undisturbed at 25°C for 30 min to allow for clotting. Both serum and citrate tubes were centrifuged at 1,710 × *g* for 10 min, which was followed by another round of centrifugation at 2,330 × *g* for 5 min. The supernatant from each tube was carefully aliquoted and stored at −80°C.

### Platelet isolation and lysate preparation

Citrate tubes were centrifuged at 90 × *g* for 15 min and the top 75% of the resultant platelet rich plasma (PRP) was carefully collected. Prostaglandin E1 (Cayman Chemical, Ann Arbor, MI) was added (final concentration: 1 μM) to inhibit platelet aggregation. PRP was recentrifuged at 2,330 × *g*, and the supernatant was completely removed to separate the platelet pellet. Platelet pellets were resuspended in PBS containing 1 μM prostaglandin E1 to an equal volume of PRP and were gently washed by mixing with a pipette. The washed platelets were recentrifuged at 2,330 × *g*, and the supernatant was completely removed. The obtained platelet pellets were lysed by resuspending in a lysis buffer (Cell Signaling Technology, Danvers, MA) containing protease inhibitors (Halt Protease Inhibitor Cocktail EDTA-free; Thermo Fisher Scientific, Waltham, MA) to equal the volume of initially adjusted PRP. The lysate was immediately stored at −80°C.

### ELISA

VEGF-A121 and VEGF-A165 were quantified using the following methods [21]: Polystyrene microtiter plates (Nunc Plate, Thermo Fisher Scientific, Waltham, MA) were coated with 100 μL of anti-human VEGF-A polyclonal antibody (R&D Systems) in PBS and incubated overnight at 4°C. The plates were washed three times with PBS containing 0.05% Tween 20, and the uncoated well binding sites were blocked with 1% BSA (400 μL/well) prepared in PBS, followed by incubation for 2 h. The plates were then washed, and 100 μL of different dilutions of the calibrator and samples (1:1 dilution in 0.2 mol/L Tris, pH 8.5, and 0.15 mol/L sodium chloride containing 1% casein) were added to the wells. The plates were incubated for 15 h at 25°C. After another round of washing, 100 μL of peroxidase-conjugated anti-human VEGF-A121 or VEGF-A165 peptide monoclonal antibody was added to each well and the plates were incubated for 2 h at 25°C. The plates were subsequently washed and 3,3′,5,5′-tetra-methylbenzidine (Dojindo Laboratories, Kumamoto, Japan) was added as a chromogenic substrate to each well. The reaction was terminated with 0.25 mol/L sodium sulfate (100 μL/well), and the absorbance at 450 nm was measured using a microplate reader (Model 680, Bio-Rad, Irvine, CA). A standard curve was drawn using recombinant human VEGF-A121 (rh VEGF-121; Cell Signaling Technology) or recombinant human VEGF-A165 (rh VEGF-165; Cell Signaling Technology), which were quantitated in a linear range of 10–2,000 pg/mL with the chromogenic substrate. Samples with absorbance above the calibration range were assayed after dilution.

VEGF-A was quantified using the Human VEGF Quantikine ELISA Kit (R&D Systems), and bevacizumab blood levels were measured using a Bevacizumab ELISA Kit (ab237642; Abcam Cambridge, MA).

### Recombinant proteins, bevacizumab, and plasmids

RhVEGF-A121 and rhVEGF-A165 proteins were purchased from Cell Signaling Technology. Bevacizumab was purchased from R&D Systems (Human VEGF Antibody, MAB9947). Expression plasmids for hVEGF-A121 (#HG10008-NF) and hVEGF-A165 (#RC229662) were purchased from Sino Biological (Beijing, China) and OriGene Technologies (Rockville, MD), respectively.

### Cell culture and transfection

HEK293 cells were purchased from American Type Culture Collection (ATCC, Manassas, VA) and cultured in high glucose Dulbecco’s Modified Eagle Medium (DMEM; Gibco, Life Technology, Grand Island, NY), supplemented with 10% FBS at 37°C in a humidified 5% CO_2_ atmosphere. Cells were transfected with hVEGF-A121 or hVEGF-A165 expression plasmids using Lipofectamine 3000 (Invitrogen, Life Technologies, Carlsbad, CA), according to the manufacturer’s instructions. Transfected cells were cultured for two days, after which the conditioned media were collected.

### Immunoprecipitation of VEGF from serum samples

The removal of human IgG (including bevacizumab) from serum samples was performed as described in previous reports [9, 10, 16, 23]. Serum (200 μL) was mixed with 100 μL of protein A/G PLUS-agarose (Santa Cruz Biotechnology, Inc., Dallas, TX) for 4 h by rotation at 4°C and then centrifuged for 5 min at 1,000 × *g*; 200 μL of the supernatant was again mixed with 100 μL of protein A/G PLUS-agarose and subjected to rotation overnight. After two consecutive centrifugation steps, the concentration of VEGF in the supernatant was analyzed via ELISA. The concentrations, thus determined, were multiplied by a factor of 1.9 to adjust for the dilution of samples in the immunoprecipitation (IP) procedure.

### Statistical analysis

Statistical analyses were performed using MATLAB R2022b (MathWorks, Natick, MA, USA) and GraphPad Prism version 9 (San Diego, CA, USA). For each duo of paired-data samples, the effect size was calculated as the median of the paired differences, the 95% confidence interval (CI) was calculated using bias-corrected and accelerated (BC_a_) bootstrapping employing 10,000 resamples with replacement, and the *p* value was calculated using the Wilcoxon Signed Rank Test. The effect size is the best estimate of the change between the underlying population, and the CI conveys the precision of this estimate. A *p* values less than 0.05 indicates a statistically significant change in population. Asterisks in figures indicate the magnitude of the *p* value (*p < 0.05, **p < 0.005, *** *p* < 0.0005); *ns* stands for no statistical significance.

## Results

### Concentrations of VEGF-A121, VEGF-A165, and VEGF-A in serum and plasma samples

VEGF-A121, VEGF-A165, and VEGF-A in serum and plasma samples were quantified before and 2–4 weeks post-bevacizumab administration using an ELISA system that can accurately distinguish between VEGF-A121 and VEGF-A165 [21], and a commercially available and widely used ELISA system (Human VEGF Quantikine ELISA Kit, R&D Systems, Minneapolis, MN) that measures VEGF-A (both VEGF-A121 and VEGF-A165).

**Fig 1:**
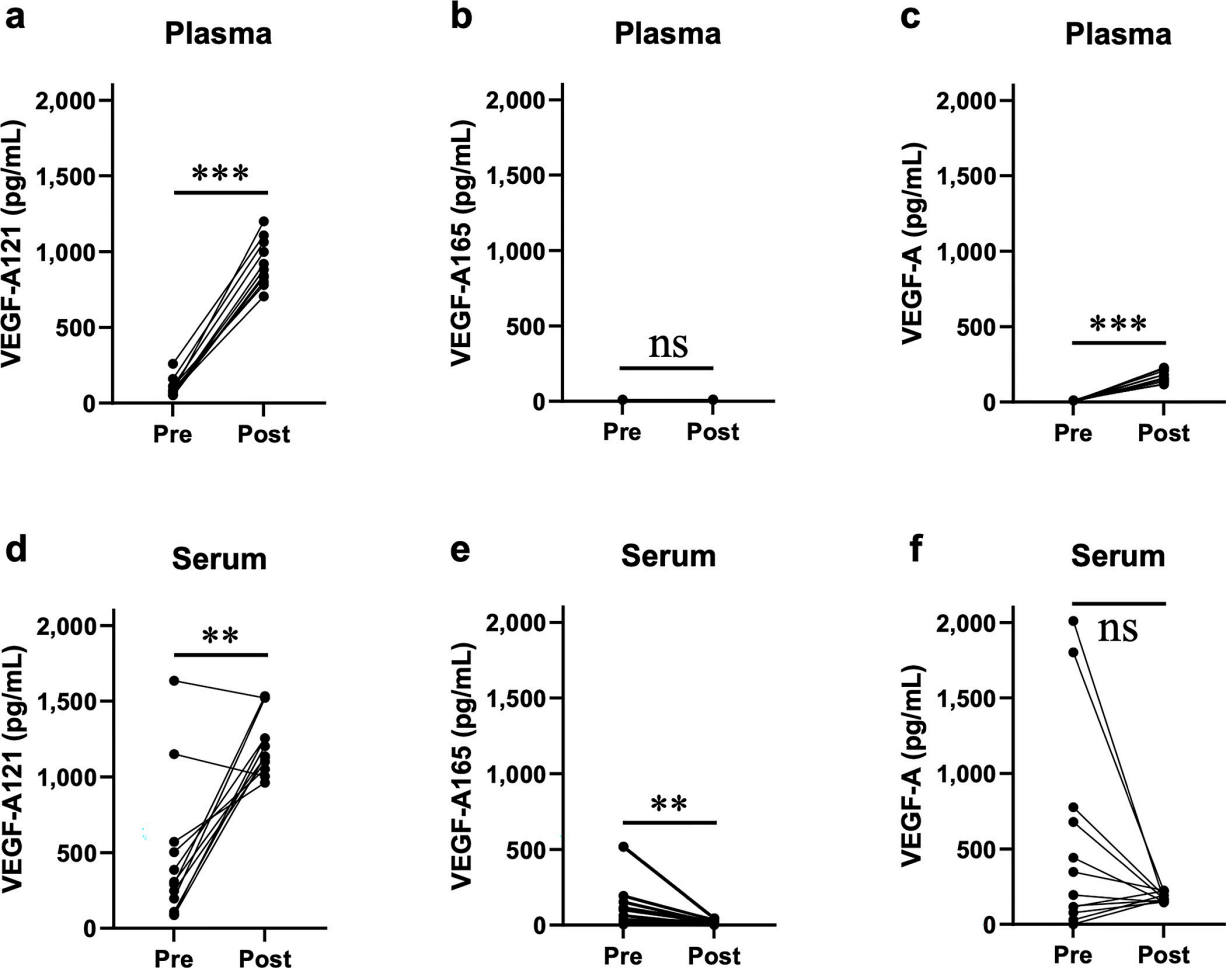
Blood concentrations of VEGF-A121, VEGF-A165, and VEGF-A pre- and post-bevacizumab administration. VEGF-A121, VEGF-A165, and VEGF-A concentrations in plasma (**a**-**c**) and in serum (**d**-**f**) were measured pre- and post-bevacizumab administration, showing that only VEGF-A121 is a clear marker of bevacizumab therapy in both plasma and serum. **a** Plasma VEGF-A121 exhibited a median increase of 808.6 pg/mL, 95% CI [748.7, 874.0], *p* = 0.00049. **b** Plasma VEGF-A165 concentration was below the high-accuracy threshold, both pre- and post-administration, and exhibited a statistically insignificant median change of 0 pg/mL, 95% CI [–1.7, –1.6], *p* = 0.3125. **c** Plasma VEGF-A exhibited a median change of 156.9 pg/mL, 95% CI [126.8, 190.0], *p* = 0.00026. **d** Serum VEGF-A121 exhibited a median increase of 860.8 pg/mL, 95% CI [468.5, 1128.9], *p* = 0.0024. **e** Serum VEGF-A165 exhibited a medium change of –73.8 pg/mL, 95% CI [–149.4, –10.2], *p* = 0.0034, with 10 of 12 (83.3%) of the post-bevacizumab concentrations falling below the high-accuracy threshold of 38 pg/mL. **f** Serum VEGF-A exhibited a median change of –86.1 pg/mL, 95% CI [–545.4, 88.74], *p* = 0.1763.

The concentration of VEGF-A121 exhibited a substantial and statistically significant increase in both the plasma and serum samples post-bevacizumab administration (Fig 1A, 1D); the median increase in serum was 860.8 pg/mL, 95% CI [468.5, 1128.9], p = 0.0024, and in plasma was 808.6 pg/mL, 95% CI [748.7, 874.0], *p* = 0.00049. For the pre and post samples in serum, the two pairs that started above 1000 pg/mL both exhibited a *decrease* in concentration (Fig 1D); their median change of –131.3 pg/mL was much weaker than the median change of 959.68 pg/mL of the other pairs. All measurements exceeded the high-accuracy threshold of 35 pg/mL.

The serum concentration of VEGF-A165 decreased post-bevacizumab administration having a medium change of –73.8 pg/mL, 95% CI [–149.4, –10.2], *p* = 0.0034, with 10 of 12 (83.3%) of the post-bevacizumab concentrations falling below the high-accuracy threshold of 38 pg/mL (Fig 1E). The VEGF-A165 plasma concentration was below the high-accuracy threshold both pre- and post-bevacizumab administration (Fig 1B) and showed a statistically insignificant median change of 0 pg/mL, 95% CI [–1.7, –1.6], *p* = 0.3125.

The concentration of VEGF-A, including both VEGF-A121 and VEGF-A165, exhibited a statistically insignificant decrease in serum of –86.1 pg/mL, 95% CI [–545.4, 88.74], *p* = 0.1763, where the concentration increased post-bevacizumab administration in five cases and decreased in seven cases (Fig 1F). In all cases in plasma, VEGF-A concentrations were increased post-bevacizumab administration (Fig 1C), with a median change of 156.9 pg/mL, 95% CI [126.8, 190.0], *p* = 0.00026. Thus, VEGF-A did not vary in a consistent manner across plasma and serum environments.

### VEGF-A121, VEGF-A165, and VEGF-A contents in platelet lysates

**Fig 2:**
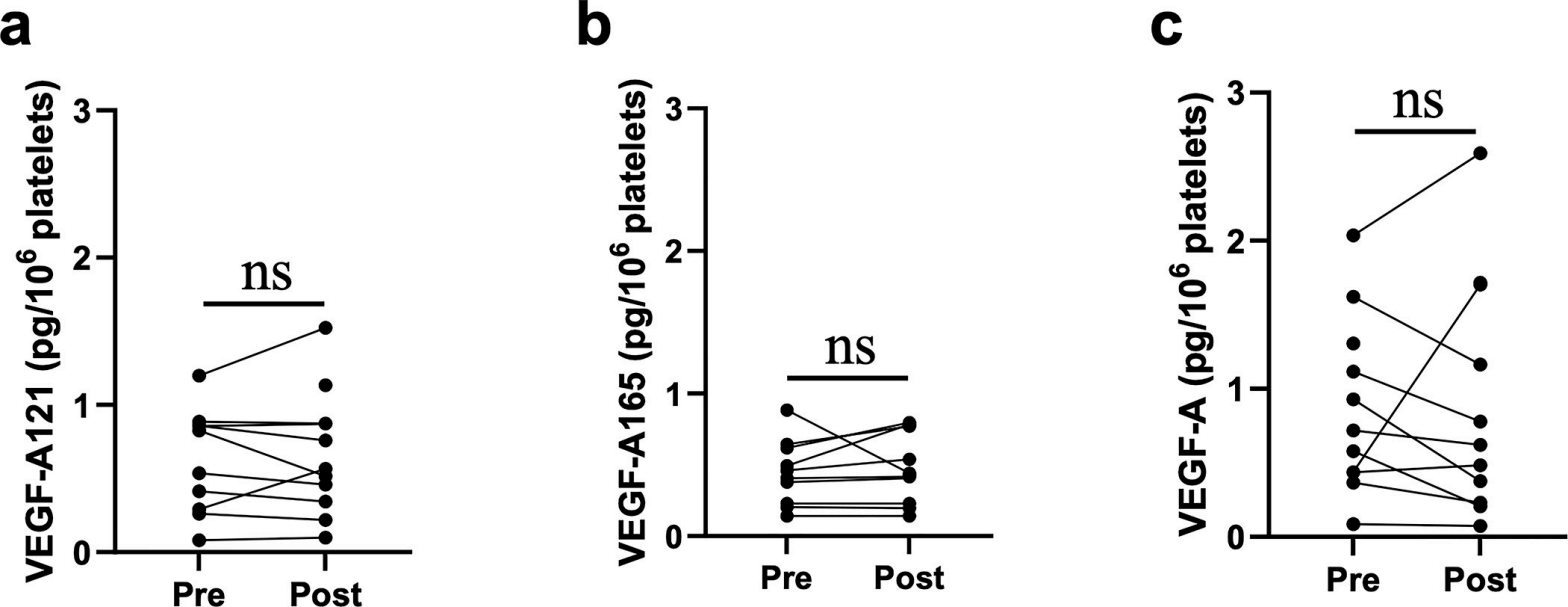
VEGF-A121, VEGF-A165, and VEGF-A contents per unit platelets. Concentrations of VEGF-A121, VEGF-A165, and VEGF-A in the platelet lysates, showing that the amounts of VEGF-A and its isoforms in platelets do not change to a statistically significant degree between pre- and post-bevacizumab administration. **a** VEGF-A121 exhibited a median change of –0.03 pg/10^6^ platelets, 95% CI [–0.08, 0.14], *p* = 0.625. **b** VEGF-A165 exhibited a median change of –0.02 pg/10^6^ platelets, 95% CI [–0.004, 0.129], *p* = 0.250. **c** VEGF-A exhibited a median change of –0.117 pg/10^6^ platelets, 95% CI [–0.374, 0.301], *p* = 0.556.

Platelet VEGF-A released upon coagulation affects VEGF-A concentrations in serum samples [23–26]. Therefore, VEGF-A concentrations in platelet lysates were analyzed before and after bevacizumab administration to assess their potential role of coagulation in the serum data of Fig 1. The amount of VEGF-A121, VEGF-A165, and VEGF-A in 10^6^ platelets were calculated by dividing the number of platelets in the PRP from which the platelet lysate was derived. The amount of VEGF-A121, VEGF-A165, and VEGF-A per 10^6^ platelets did not exhibit a statistically significant change post-bevacizumab administration (Fig 2A–2C). VEGF-A121 exhibited a median change of –0.03 pg/10^6^ platelets, 95% CI [–0.08, 0.14], *p* = 0.625, VEGF-A165 exhibited a median change of –0.02 pg/10^6^ platelets, 95% CI [–0.004, 0.129], *p* = 0.250, and VEGF-A exhibited a median change of –0.117 pg/10^6^ platelets, 95% CI [–0.374, 0.301], *p* = 0.556.

### Bevacizumab concentration in serum

The concentration of bevacizumab in serum pre and post its administration was measured using ELISA; pre administration, the concentration of bevacizumab was below the limit of detection, while post administration, the concentration (mean ± SD) was 26.8 ± 11.7 μg/mL.

### *In vitro* neutralization experiments with VEGF-A and bevacizumab

**Fig 3:**
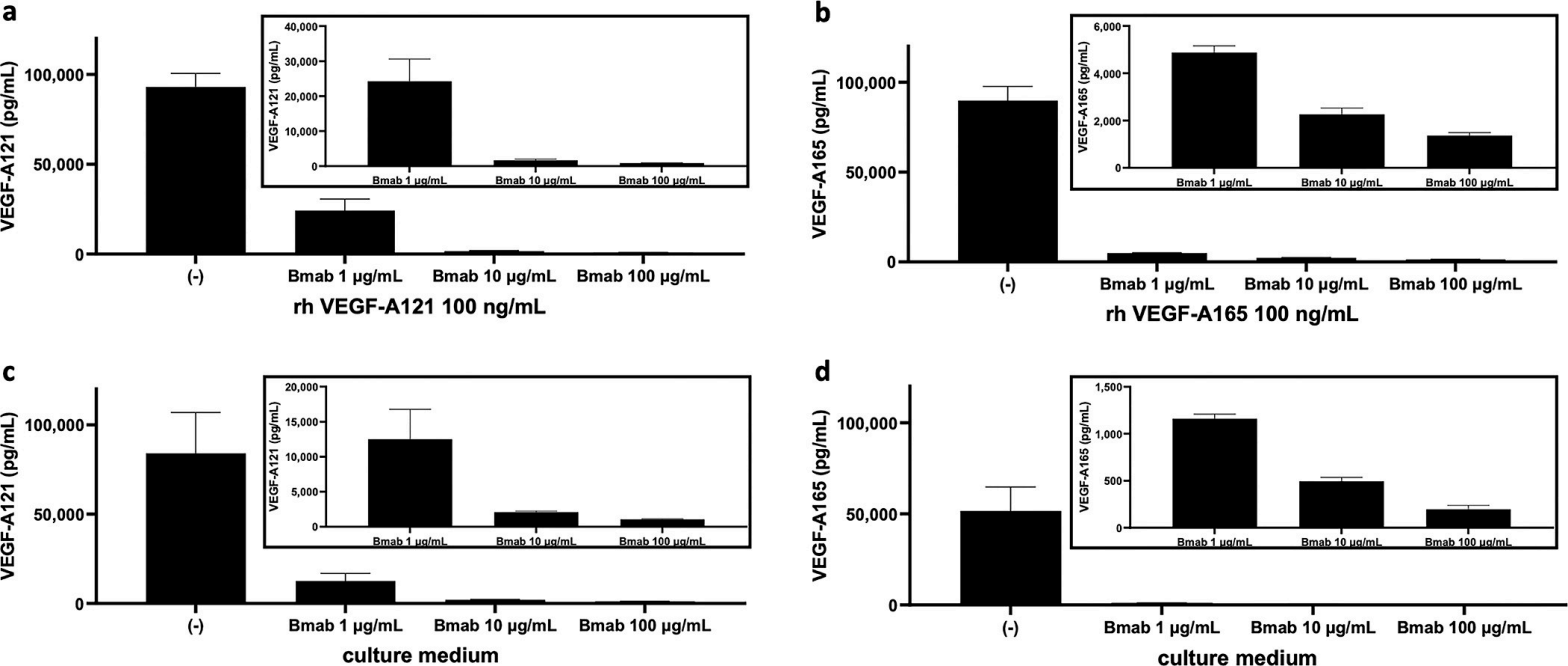
Effects of bevacizumab addition on VEGF-A concentrations *in vitro*. Bevacizumab absorbed VEGF-A121 and VEGF-A165 in a dose dependent manner. **a** and **b** show the results of bevacizumab addition to rhVEGF-A. **a** Bevacizumab was added at 1, 10, and 100 μg/mL to rhVEGF-A121, adjusted to a concentration of 100 ng/mL. After the one-way ANOVA test, a test of linear trend between column means and column order from left to right was performed for multiple comparisons, and a linear trend was observed between VEGF-A121 and bevacizumab concentrations (ANOVA: *p* < 0.0001; test for trend: *p* < 0.0001). **b** Similar experiments were performed using VEGF-A165 (ANOVA: *p* < 0.0001; test for trend: *p* < 0.0001). **c** and **d** show the results of bevacizumab addition to the culture medium of HEK293 cells transfected with VEGF-A. **c** HEK293 cells were transfected with VEGF-A121 and cultured in a medium containing 1, 10, or 100 μg/mL bevacizumab, and in a culture medium without bevacizumab (control). The culture medium was collected, and VEGF-A121 concentrations were measured. The same test was performed, and a linear trend was observed (ANOVA, *p* < 0.0001; test for trend, *p* < 0.0001). **d** The same experiment was performed for VEGF-A165 (ANOVA: *p* < 0.0001; test for trend: *p* < 0.0001).

The neutralization of VEGF-A antigen by bevacizumab was tested *in vitro*. Bevacizumab (at 1, 10, and 100 μg/mL—concentrations close to that in the blood described above) was added to 100 ng/mL rhVEGF-A121 and rhVEGF-A165 solutions and the mixture was incubated overnight at 4°C. The adjusted antigen concentration of 100 ng/mL was markedly reduced by the addition of bevacizumab (Fig 3A and 3B); the concentrations of VEGF-A121 and VEGF-A165 decreased with increasing bevacizumab concentration (Fig 3A and 3B, inset). Neutralization experiments were performed by adding bevacizumab in cultured cell media. HEK293 cells were transfected with VEGF-A121 or VEGF-A165 expression plasmids and cultured for two days. After washing, the cells were replaced with a medium containing known concentrations of bevacizumab (1, 10, and 100 μg/mL) or without bevacizumab and cultured overnight at 37°C, and the conditioned medium was collected and analyzed. VEGF-A121 and VEGF-A165 in the culture supernatant were neutralized with bevacizumab. VEGF-A121 and VEGF-A165 concentrations decreased in proportion to the concentration of bevacizumab (Fig 3C and 3D). These results suggest that measurement of VEGF-A121 or VEGF-A165 does not detect a complex of bevacizumab and VEGF-A121 or bevacizumab and VEGF-A165, respectively.

### Measurement of plasma samples neutralized with residual bevacizumab by adding rhVEGF-A

**Fig 4:**
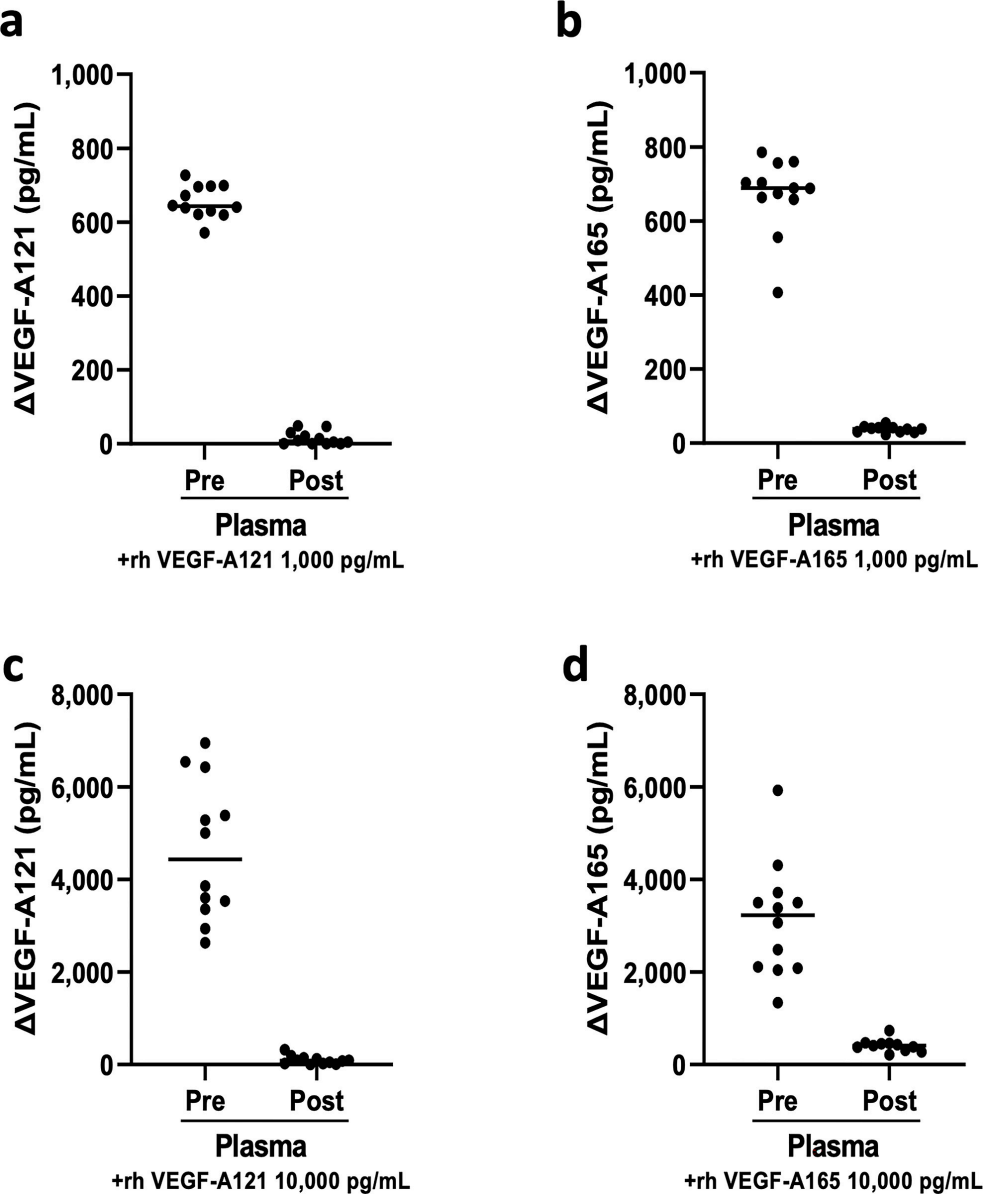
Effect of adding rhVEGF-A (-121 or -165) on the concentrations of VEGF-A121 and VEGF-A165 in plasma samples. Addition of rhVEGF-A (-121 or-165; 1,000 pg/mL or 10,000 pg/mL) to plasma samples pre- and post-bevacizumab administration, and the magnitude of change associated with the addition was calculated by subtracting the concentration in plasma samples without rhVEGF-A from the concentration in plasma samples with rhVEGF-A added. **a** Plasma samples pre-bevacizumab administration showed an increase in VEGF-A121 concentration (mean ± SD: 655 ± 44 pg/mL) corresponding to the addition of 1,000 pg/mL of rhVEGF-A121; plasma samples post-bevacizumab administration showed no increase in the concentration corresponding to the addition (mean ± SD: 15 ± 18 pg/mL). **b** Similarly, the addition of 1,000 pg/mL of rhVEGF-A165 increased the concentration of VEGF-A165 in plasma samples pre-bevacizumab administration (mean ± SD: 670 ± 102 pg/mL), whereas the increase in concentration was negligible in plasma samples post-bevacizumab administration (mean ± SD: 38 ± 9 pg/mL). **c** Addition of 10,000 pg/mL rhVEGF-A121 increased the concentration of VEGF-A121 in plasma samples pre-bevacizumab administration (mean ± SD: 4,624 ± 1,500 pg/mL), whereas the increase in concentration was negligible in plasma samples post-bevacizumab administration (mean ± SD: 96 ± 92 pg/mL). **d** Addition of 10,000 pg/mL rhVEGF-A165 increased the concentration of VEGF-A165 in plasma samples pre-bevacizumab administration (mean ± SD: 3,121 ± 1,236 pg/mL), whereas the increase in concentration was negligible in plasma samples post-bevacizumab administration (mean ± SD: 409 ± 134 pg/mL).

To investigate the neutralizing effect of residual bevacizumab, recombinant VEGF-A was added to the plasma samples pre- and post-bevacizumab administration. Plasma samples were incubated overnight with or without rhVEGF-A121 and rhVEGF-A165 (1,000 or 10,000 pg/mL) at 4°C. The amount of change because of recombinant VEGF-A addition was calculated by subtracting the concentration in plasma samples without recombinant VEGF-A from the concentration in plasma samples with recombinant VEGF-A. The concentrations of VEGF-A121 and VEGF-A165 increased with the amount of VEGF-A added to the plasma samples pre-bevacizumab administration (mean ± SD; VEGF-A121 1,000 pg/mL: 655 ± 44 pg/mL; VEGF-A165 1,000 pg/mL: 670 ± 102 pg/mL; VEGF-A121 10,000 pg/mL: 4,624 ± 1,500 pg/mL; VEGF-A165 10,000 pg/mL: 3,121 ± 1,236 pg/mL), but not in the plasma samples post-bevacizumab administration (VEGF-A121 1,000 pg/mL: 15 ± 18 pg/mL; VEGF-A165 1,000 pg/mL: 38 ± 9 pg/mL; VEGF-A121 10,000 pg/mL: 96 ± 92 pg/mL; VEGF-A165 10,000 pg/mL: 409 ± 134 pg/mL) (Fig 4A–4D). Thus, in plasma samples prior to bevacizumab administration, the majority of added VEGF-A was absorbed by bevacizumab.

### Experimental neutralization of VEGF-A by addition of bevacizumab to serum samples

**Fig 5:**
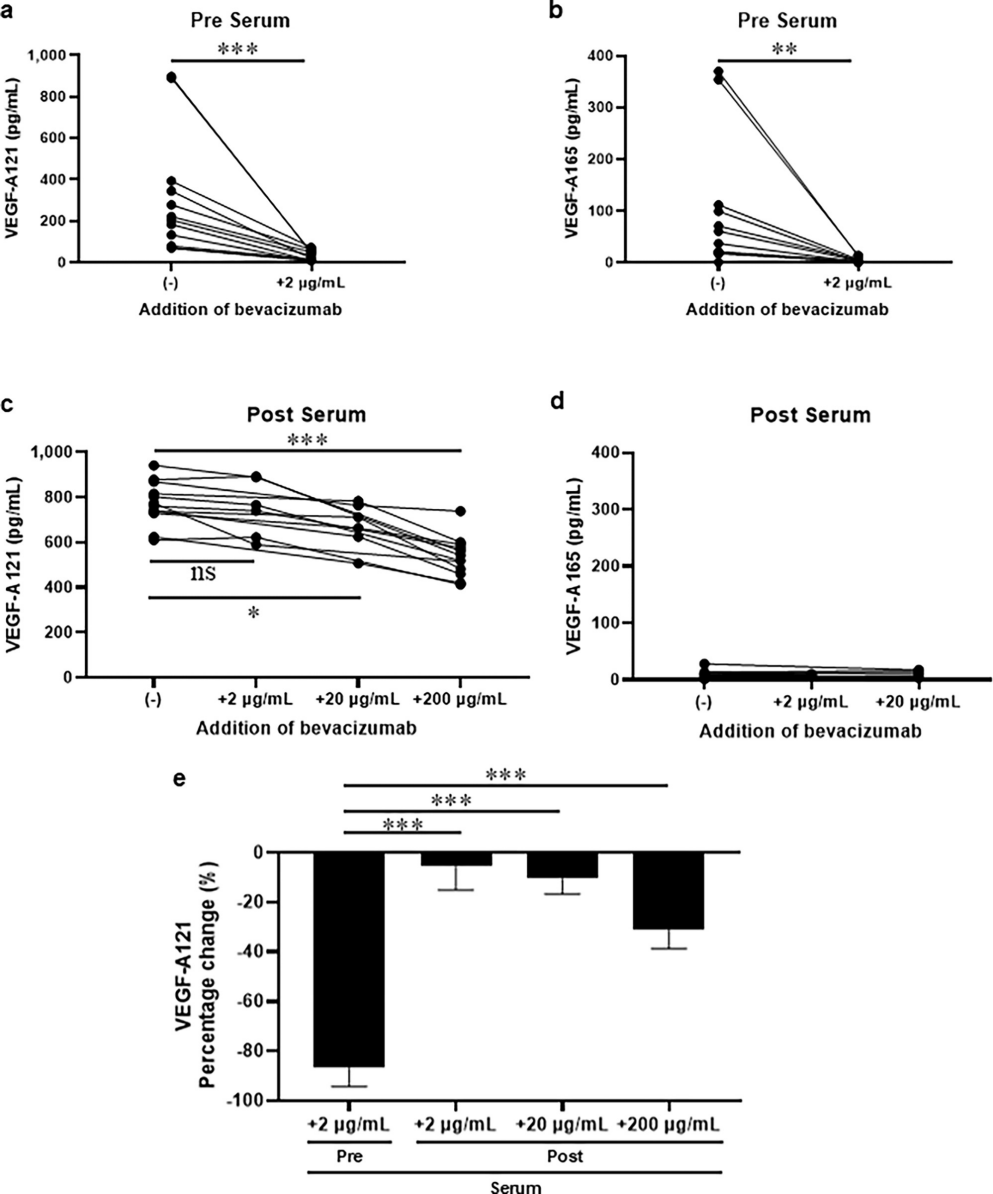
Neutralization with additional bevacizumab to serum samples pre- and post-bevacizumab administration. Serum samples pre-bevacizumab administration were treated with or without bevacizumab (2 μg/mL). Serum samples after bevacizumab treatment with an additional 2, 20, or 200 μg/mL of bevacizumab were compared to control samples without bevacizumab, indicating that free VEGF-A121 remained even at high doses of bevacizumab addition. **a** VEGF-A121 exhibited a change of –173.2, 95% CI [–322.8, –96.3], *p* = 0.00049, where the addition of 2 μg/mL bevacizumab markedly reduced the VEGF-A121 concentration to a median of 26.9 pg/mL, with a range of 67.3 pg/mL that partly fell below the high-accuracy threshold of 38 pg/mL. **b** VEGF-A165 concentrations exhibited a change of –46.4, 95% CI [–101.4, –16.6], *p* = 0.0015, driven down to a median of 2.9 pg/mL with a range of 14.12 pg/mL that is entirely below the high-accuracy threshold. **c** Further addition of bevacizumab to serum samples post-bevacizumab administration significantly decreased VEGF-A121 levels in samples to which 20 and 200 μg/mL bevacizumab was added (ANOVA: *p* < 0.0001; Dunnett’s multiple comparisons test: +2 μg/mL, *p* = 0.7096 vs control; +20 μg/mL, *p* = 0.0107 vs control; +200 μg/mL, *p* < 0.0001 vs control). **d** VEGF-A165 concentrations were low in serum samples post-bevacizumab administration and remained low after further addition of bevacizumab. **e** The percent change in VEGF-A121 concentration with the addition of any (2, 20, or 200 μg/mL) concentration of bevacizumab to serum samples post-bevacizumab administration was significantly less than the percent change in VEGF-A121 concentration with the addition of 2 μg/mL concentration of bevacizumab to serum samples pre-bevacizumab administration (ANOVA: *p* < 0.0001; Dunnett’s multiple comparisons test: Post+2 μg/mL, *p* < 0.0001 vs Pre+2 μg/mL; Post +20 μg/mL, *p* < 0.0001 vs Pre+2 μg/mL; +200 μg/mL, *p* < 0.0001 vs Pre+2 μg/mL).

Additional bevacizumab was added to serum samples pre- and post-bevacizumab administration to determine its neutralizing effect. Serum samples were rotated overnight at 4°C with bevacizumab concentration adjusted in PBS and an equal volume of PBS as control, and VEGF-A121 and VEGF-A165 were measured. Both VEGF-A121 and VEGF-A165 showed a statistically significant decreased in serum samples prior to bevacizumab treatment with bevacizumab at 2 μg/mL compared with serum samples without bevacizumab (Fig 5A and 5B). VEGF-A121 exhibited a change of –173.2, 95% CI [–322.8, –96.3], *p* = 0.00049, whereas VEGF-A165 exhibited a change of –46.4, 95% CI [–101.4, –16.6], *p* = 0.0015. The neutralizing impact of 2 μg/mL of bevacizumab drove the median VEGF-A165 down to 2.9 pg/mL, with a range of 14.12 pg/mL that is entirely below the high-accuracy threshold of 38 pg/mL. VEGF-A121 was driven to a median of 26.9 pg/mL, with a 67.3 pg/mL range that partially fell below the high-accuracy threshold of 35 pg/mL. Serum samples, post-bevacizumab treatment, were supplemented with an additional 2 μg/mL (1/10 of the remaining serum bevacizumab concentration), 20 μg/mL (equivalent to the remaining serum bevacizumab concentration), and 200 μg/mL (10 times the remaining serum bevacizumab concentration). VEGF-A121 showed a tendency to decrease in proportion to the added bevacizumab (Fig 5C); VEGF-A165 was low in the serum samples post-bevacizumab treatment without further addition of bevacizumab and remained unaltered by the addition of bevacizumab (Fig 5D). Even higher bevacizumab doses (200 μg/mL) added to serum samples post-bevacizumab administration had a smaller neutralizing effect than addition of lower bevacizumab (2 μg/mL) to serum samples pre-bevacizumab administration (Fig 5E).

### Measurement of serum samples with VEGF-A–bevacizumab complexes removed using immunoprecipitation

**Fig 6:**
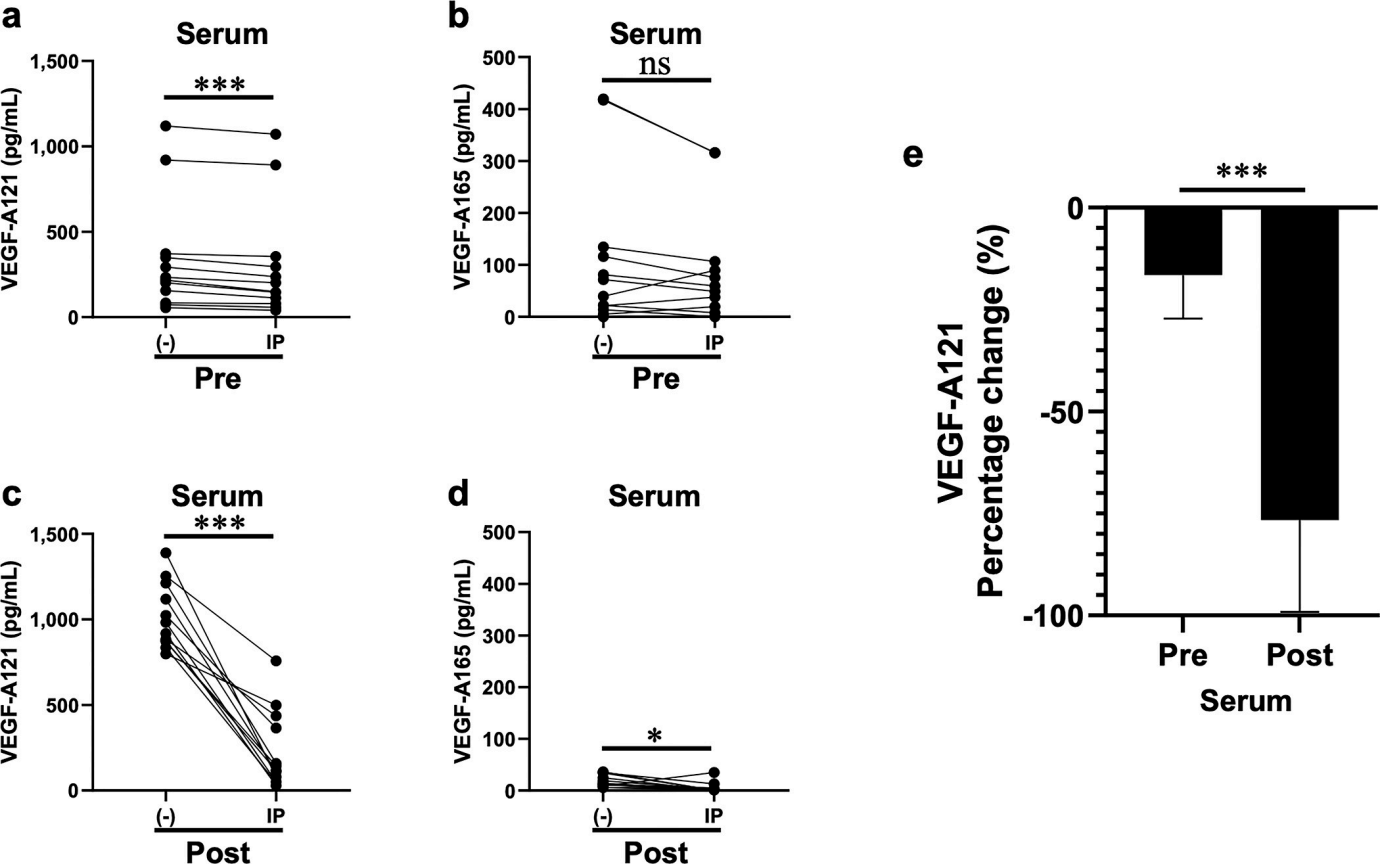
Effect of the VEGF-A–bevacizumab complex removal using immunoprecipitation on the concentrations of VEGF-A121, VEGF-A165, and VEGF-A. Serum samples pre- and post-bevacizumab administration were immunoprecipitated to remove the VEGF-A–bevacizumab complex and the isoform concentrations were measured in the remaining serum samples, showing that VEGF-A121 post administration exhibited the most substantial change. **a** Pre-administration, VEGF-A121 exhibited a median change of –37.3 pg/mL, 95% CI [–54.6, –16.0], *p* = 0.00049. **b** Pre-administration, VEGF-A165 exhibited a statistically insignificant median change of –18.1 pg/mL, 95% CI [–34.2, 7.5], *p* = 0.1475 **c** Post-administration, VEGF-A121 exhibited a substantial median change of –771.8 pg/mL, 95% CI [–970.9, –567.5], *p* = 0.00049. **d** Post-administration VEGF-A165 exhibited a small median change of –12.5 pg/mL, 95% CI [–23.4, –6.1], *p* = 0.0210. **e** Due to the possibility of nonspecific removal of VEGF-A using immunoprecipitation, the rate of change in VEGF-A121 concentration due to immunoprecipitation was compared pre- and post-bevacizumab administration and was reduced more by immunoprecipitation in specimens post-bevacizumab administration.

VEGF-A concentration was reportedly decreased in blood samples from which the VEGF-A–bevacizumab complex was removed via immunoprecipitation [9, 10, 16, 23]; this was further assessed in the present study. The concentrations of VEGF-A121 in the serum remaining following immunoprecipitation of serum samples pre-bevacizumab administration showed a statistically significant although moderately small decrease with a median change of –37.3 pg/mL, 95% CI [–54.6, –16.0], *p* = 0.00049 (Fig 6A). The associated VEGF-A165 concentration showed a median change of –18.1 pg/mL, 95% CI [–34.2, 7.5], *p* = 0.1475 (Fig 6B); this high p-value does not provide sufficient evidence to reject the null hypothesis. Immunoprecipitation of serum samples post-bevacizumab administration resulted in a statistically significant decrease in the concentrations of VEGF-A121 and VEGF-A165 in the remaining serum (Fig 6C and 6D), although to different degrees. VEGF-A121 showed a substantial median change of –771.8 pg/mL, 95% CI [–970.9, –567.5], *p* = 0.00049 (Fig 6C), whereas VEGF-A165 showed a small median change of –12.5 pg/mL, 95% CI [–23.4, –6.1], *p* = 0.0210 (Fig 6D). The average percentage change caused by immunoprecipitation of serum samples post-bevacizumab administration was −76.6%, which was more marked than that in samples pre-bevacizumab administration (Fig 6E). Note that the obtained values were multiplied by 1.9 to correct for the effect of dilution during immunoprecipitation.

## Discussion

There have been several reports on the measurement of blood concentrations of VEGF-A in patients treated with bevacizumab, yet a consensus on whether these concentrations decrease [9–11] or increase [9, 12–17] post-bevacizumab administration remains lacking. Decreased VEGF-A concentrations have been reported for blood samples taken immediately post-bevacizumab administration or in cases where blood samples were subjected to immunoprecipitation for the removal of the VEGF-A–bevacizumab complex and concentration of free VEGF-A was measured [9–11]. Reports of increased VEGF-A concentrations were obtained for blood samples collected on days 3–21 post-bevacizumab administration, without removal of the complex by immunoprecipitation [9, 12–17]. In the present study, blood samples were collected on days 14 through 28 post-bevacizumab administration. VEGF-A was found to increase with statistically significant median change of 156.9 pg/mL in plasma but found to decrease with statistically insignificant median change of –86.1 pg/mL in serum (Fig 1C and 1F). The net inconsistency across the literature of VEGF-A behavior motivates our investigation into changes in the VEGF-A isoforms.

**Fig 7:**
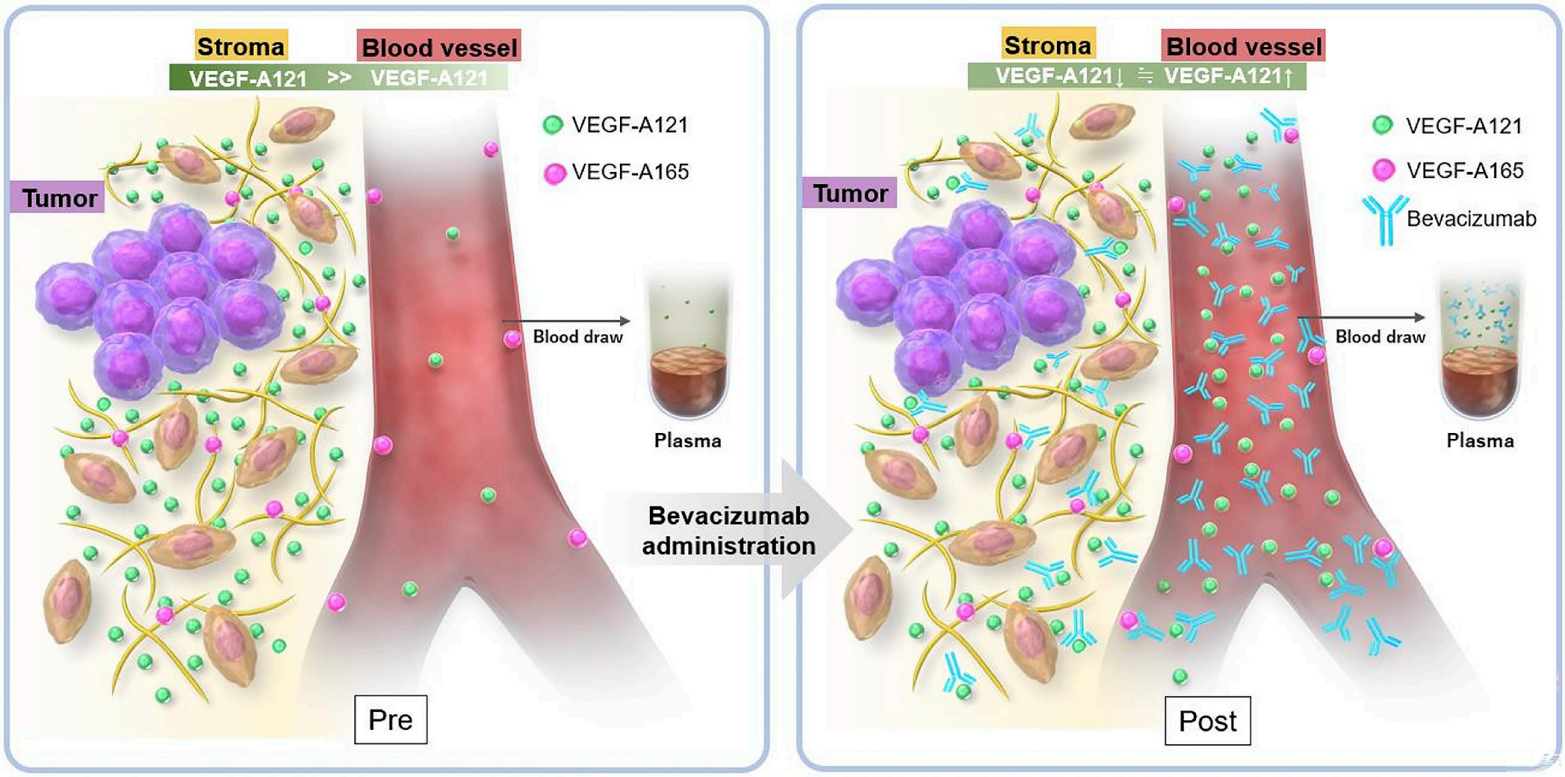
Hypothetical mechanism by which plasma VEGF-A121 concentration is elevated post-bevacizumab administration. The left panel shows the condition pre-bevacizumab administration. VEGF-A121 is present at high concentrations in the tumor stroma; VEGF-A165 binds to the interstitial matrix and vessel walls for binding to HSPG. Right panel shows the condition post-bevacizumab administration. Free VEGF-A121 in the tumor stroma and free VEGF-A121 in the plasma reach an equal concentration; thus, the plasma VEGF-A121 concentration increases and the VEGF-A121 concentration in the tumor stroma decreases.

A marked and statistically significant increase in VEGF-A121 concentrations was observed in plasma and serum samples (Fig 1A and 1D), exhibiting a median increase of 808.6 and 860.8 pg/mL, respectively. VEGF-A165 concentrations in plasma samples were below the limit of detection pre- and post-bevacizumab administration (Fig 1B), which indicates that the increase in VEGF-A concentration in plasma reflects the increase in VEGF-A121. VEGF-A121 lacks the heparan sulfate proteoglycan binding sites, exons 6 and 7, and can diffuse more freely into the circulating blood and interstitial fluid than other isoforms [19]. In pharmacokinetic models, assuming that bevacizumab leaks out of the blood vessels, the "diffusible" VEGF-A molecules move from high concentrations in the interstitial fluid to low concentrations in the circulating blood, and the concentration of free VEGF-A (bevacizumab-unbound VEGF-A) in the circulating blood remains approximately 9-fold higher than baseline even 3 weeks post-bevacizumab administration [27, 28]. Therefore, the increase in plasma VEGF-A121 concentration may be attributed to the marked increase in free VEGF-A121 in the circulating blood due to the transfer of diffusible VEGF-A121 from the interstitial fluid into the circulating blood post-bevacizumab administration (Fig 7). The concentration of VEGF-A165 in plasma was below the high-accuracy threshold in all cases, both pre- and post-bevacizumab administration (Fig 1B). VEGF-A165 is not a major isoform in plasma, which is the liquid phase of circulating blood since it has exon 7 and binds to substrates and cell membrane surfaces [22].

The concentration of VEGF-A165 in serum exhibited a statistically significant decrease with a median change of –73.8 pg/mL (Fig 1E). Platelets are one of the major storages of VEGF-A and serum VEGF-A is derived from activated platelets [24–26, 29, 30] (Fig 1E). The contents of VEGF-A165 in the platelets did not change pre- and post-bevacizumab administration (Fig 2B), which indicates that VEGF-A165 released from platelets due to the activation of coagulation may have been quickly neutralized when a sufficient amount of antibody remained. Post-bevacizumab administration, 26.8 μg/mL of it remained in the serum, which is similar to the concentration reported in the drug interview form [31]. *In vitro* validation also confirmed that recombinant VEGF-A was neutralized when added to the plasma post-bevacizumab administration (Fig 4A–4D). The cases in which serum VEGF-A concentrations decreased pre- and post-bevacizumab administration were those in which serum VEGF-A165 concentration was high pre-bevacizumab administration (S1 Fig) and decreased post-bevacizumab administration; the total VEGF-A concentration in these cases may reflect the concentration of VEGF-A165.

Since VEGF-A concentrations decrease when antigen–antibody complexes are removed using immunoprecipitation, some reports indicate that increased VEGF-A concentrations can be measured using the antigen–antibody complexes [9, 10, 16, 23]. Similarly, when serum samples were immunoprecipitated to remove the complexes, VEGF-A121 and VEGF-A165 concentrations decreased in a statistically significant manner in the samples post-bevacizumab administration (Fig 6C and 6D), similar to that in previous studies on VEGF-A quantitation [9, 10, 16, 23]. However, this change occurred to different degrees—the median changes for VEGF-A121 and VEGF-A165 were –771.8 and –12.4 pg/mL, respectively. A moderately small decrease in VEGF-A121 concentration was observed in samples pre-bevacizumab administration, characterized by a median change of –37.3 pg/mL. Although the calculations were corrected for the effect of dilution, the moderately small decrease in VEGF-A concentration appeared to be due to a dilution effect or some nonspecific reason. The technique to remove antigens from the serum by immunoprecipitation may be in a nonspecific manner. It is evident that immunoprecipitation reduced the VEGF-A concentrations to a greater extent in post-bevacizumab samples (Fig 6E). However, these reports quantified only the residual VEGF-A in samples in which immunoprecipitation was performed twice with a sufficient amount of immunoprecipitation beads and residual antibodies and antigen-antibody complexes were removed as much as possible (the concentration of bevacizumab in the samples after immunoprecipitation was below the detection sensitivity in all cases), and the amount of VEGF-A in the removed complexes was not quantified. Thus, further validation is needed for quantification of VEGF-A in the removed complexes.

The average bevacizumab concentration in serum post-bevacizumab administration was 26.8 μg/mL; the molecular weight of bevacizumab is 149,000 hence the average molar concentration bevacizumab post-bevacizumab administration was approximately 2 x 10^−7^ M. The equilibrium dissociation constant K_d_ for VEGF-A121 and bevacizumab is 2.2 nM [28], which theoretically means that the VEGF-A121–bevacizumab complex is present approximately 100 times more than free VEGF-A121 (S2 Fig). *In vitro* experiments wherein VEGF-A was neutralized with bevacizumab, the concentrations of VEGF-A121 and VEGF-A165 decreased with increasing concentrations of bevacizumab. The similar concentration of bevacizumab with that in the patient’s blood (10–100 μg/mL) sufficiently reduced VEGF-A121 and VEGF-A165 from 50,000–100,000 pg/mL to approximately 1,000 pg/mL, which is the same level of patient’s VEGF-A concentration (Fig 3). This indicates that the measured concentration of VEGF-A 121 and VEGF-A 165 decreases as the complex increases, and the amount of VEGF-A121 and VEGF-A165 in the complex is much higher than free VEGF-A121 and VEGF-A165. Furthermore, the addition of bevacizumab to serum samples prior to bevacizumab administration at a concentration of 2 μg/mL (1/10 of the serum bevacizumab concentration) had a neutralizing effect on VEGF-A121 and VEGF-A165 (Fig 5A and 5B), whereas the addition of bevacizumab to serum samples post-bevacizumab administration at 2 or 20 μg/mL (similar to serum concentration) or 200 μg/mL (10 times the serum bevacizumab concentration) had a weak neutralizing effect (Fig 5E). Thus, in the presence of high concentrations of bevacizumab, VEGF-A121 is in equilibrium with the antigen-antibody reaction also the VEGF-A121-bevacizumab complex is not measured. We conclude that current ELISA can specifically measure free VEGF-A121 and the VEGF-A121- bevacizumab complex is much more abundant than free VEGF-A121.

The importance of host-derived VEGF-A regulation in the tumor stroma has been reported [32, 33]. Stefanini et al. reported that the pharmacological effect of bevacizumab is the depletion of VEGF-A in the stroma due to bevacizumab-induced transfer of diffusible VEGF-A from the stroma with high concentrations of VEGF-A to the circulating blood [28]. Kazemi et al. showed that VEGF-A121 in the tumor stroma is involved in vascular dysplasia and tumor promotion, and that inhibition of VEGF-A121 may have an antitumor effect [21]. The mechanism hypothesized in this study for the increase in plasma concentration of VEGF-A121, but not of VEGF-A165, post-bevacizumab administration is the transfer of ‘diffusible” VEGF-A121 from the stroma into the circulating blood, and the reduction in the stromal concentration of VEGF-A121 may be the central pharmacological effect of bevacizumab (Fig 7). Further case series and *in vivo* studies are needed to elucidate these mechanisms and to apply quantitation methods that can specifically measure the concentrations of VEGF-A121 and VEGF-A165 as biomarkers in clinical practice.

## Conclusion

The concentration of VEGF-A121, but not of VEGF-A165, increased post-bevacizumab administration in patients with advanced colorectal cancer. The marked increase in the median change in VEGF-A121 in serum was not reflected in the median change in VEGF-A. Thus, the VEGF-A121 isoform has been uniquely demonstrated as a clear marker of bevacizumab therapy in both plasma and serum, motivating further research on pursuing these isoforms as biomarkers in cancer care, and underscoring the importance of distinguishing between VEGF-A121 and VEGF-A165 instead of determining the concentration of VEGF-A using conventional ELISA.

## Limitation

The sample size of advanced-stage colorectal cancer patients was limited to 12 in our observational study because of patient availability, and was not determined by a pre-study power analysis. However, the substantial effect size dissimilarities found between VEGF-A121, VEGF-A165, and VEGF-A show promise for VEGF-A121 as a biomarker and motivate further research with larger subject pools. Indeed, the effect sizes provided by this observational study can be used in the pre-study power analyses of future randomized control trials involving VEGF-A121.

The lack of a control group having patients who never experienced bevacizumab treatment means that our research study is an observational study of the first-time measurement of VEGF-A isoform change using our new assay for patients undergoing bevacizumab treatment. Our observational result can be further refined and interpreted by future RCT studies that include a control group of advanced colon cancer patients that go untreated or treated without bevacizumab.

## Supporting information

S1 Fig Comparison of serum VEGF-A165 concentrations pre-bevacizumab administration in patients with increased VEGF-A post-bevacizumab administration and those with decreased VEGF-A. VEGF-A165 concentrations pre-bevacizumab administration were significantly higher in patients with reduced VEGF-A concentrations post-bevacizumab administration (Unpaired *t-*test: *p* = 0.0343). *p < 0.05.
(TIFF)

S2 Fig Equation of the equilibrium dissociation constant of the antigen-antibody reaction. K_d_ represents the equilibrium dissociation constant. [VEGF-A], [bevacizumab], and [VEGF-A-bevacizumab] represent the molar concentrations of free VEGF-A, free bevacizumab, and the VEGF-A-bevacizumab complex, respectively. If the K_d_ of VEGF-A121 is 2.2 nM and [bevacizumab] is approximately 2 × 10^−7^ M, the VEGF-A121-bevacizumab complex will increase by approximately a 100-fold compared with free VEGF-A121.
(TIFF)

S1 File The original data for the figures. All calculated data is based on the numbers in this file.
(XLSX)
