# Supplementary figures and images for: Plasma and serum concentrations of VEGF-A121, but not of VEGF-A165, increase post-bevacizumab administration
Source: PLoS One. 2024 Dec 19;19(12):e0316035. doi: 10.1371/journal.pone.0316035 (PMC11658504; doi:10.1371/journal.pone.0316035)

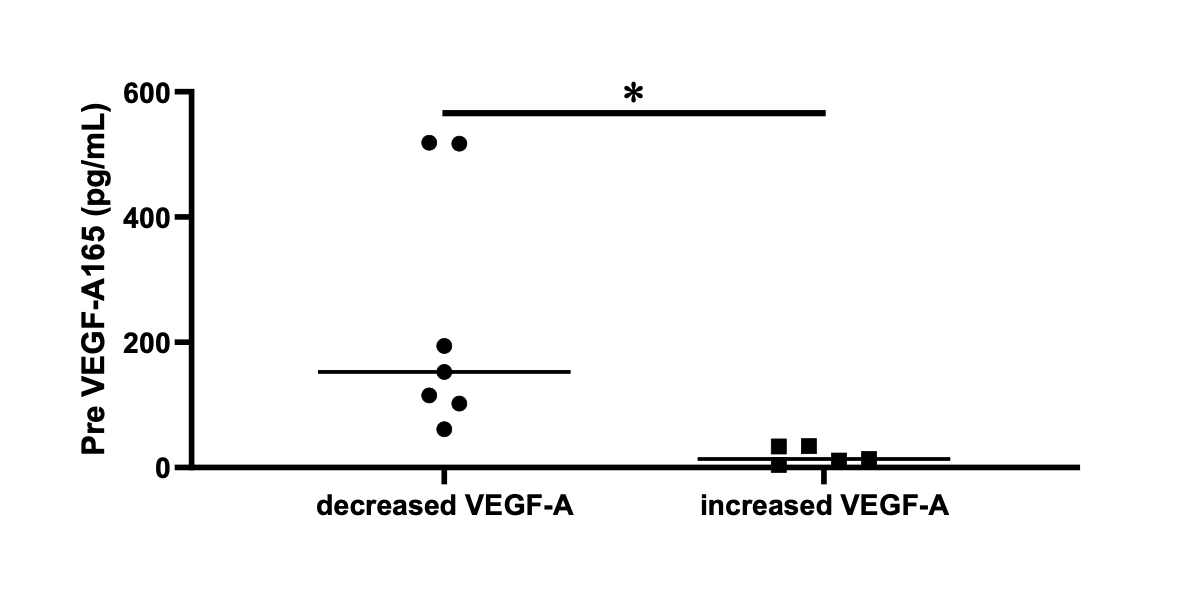

Supplement: S1 Fig — VEGF-A165 concentrations pre-bevacizumab administration were significantly higher in patients with reduced VEGF-A concentrations post-bevacizumab administration (Unpaired t-test: p = 0.0343). *p < 0.05. (TIFF) [file pone.0316035.s001.tiff]

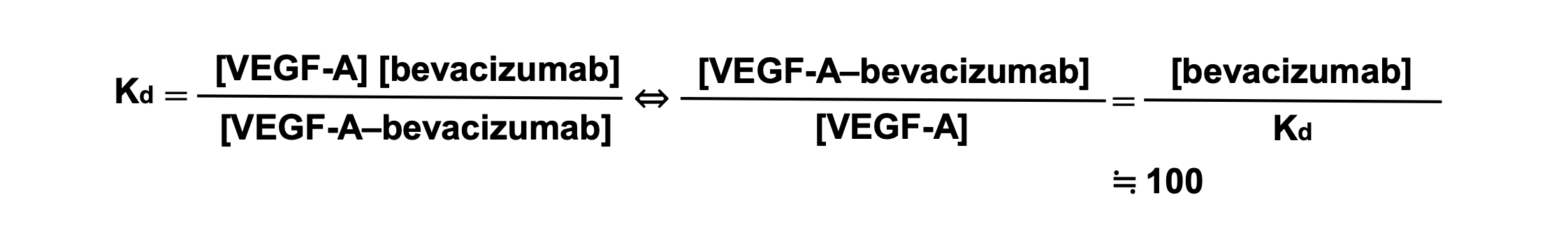

Supplement: S2 Fig — Kd represents the equilibrium dissociation constant. [VEGF-A], [bevacizumab], and [VEGF-A-bevacizumab] represent the molar concentrations of free VEGF-A, free bevacizumab, and the VEGF-A-bevacizumab complex, respectively. If the Kd of VEGF-A121 is 2.2 nM and [bevacizumab] is approximately 2 × 10^−7^ M, the VEGF-A121-bevacizumab complex will increase by approximately a 100-fold compared with free VEGF-A121. (TIFF) [file pone.0316035.s002.tiff]
